# Generational Succession of Rectal Microbiome and Metabolome in Holstein–Norwegian Red Crossbred Offspring and Their Regulatory Effects on Milk Quality

**DOI:** 10.3390/microorganisms14102169

**Published:** 2026-09-25

**Authors:** Xingyuan Wang, Wei Huang, Yuzhu Sha, Pengyang Shao, Qianling Chen, Yapeng He, Dingshan Chen, Weitao Dong, Xiu Liu, Yong Zhang

**Affiliations:** 1 College of Veterinary Medicine, Gansu Agricultural University, Lanzhou 730070, China; song1846800497@163.com (X.W.); dongwt@gsau.edu.cn (W.D.); 2 Animal Disease Prevention and Control Center of Qilihe District, Lanzhou 730050, China; 3 College of Animal Science and Technology, Gansu Agricultural University, Gansu Provincial Technical System for Modern Cold-Arid Characteristic Agricultural Cattle Industry, Lanzhou 730070, China; 18294737108@163.com (W.H.); 18894314093@163.com (Y.S.); shaopengyang666@163.com (P.S.); chenqianling223@163.com (Q.C.); 18894448066@163.com (Y.H.); 19193118787@163.com (D.C.)

**Keywords:** Holstein crossbred dairy cows, generational succession, rectal microbiome, metabolome, milk quality

## Abstract

This study aimed to explore the generational succession rules of rectal microbes and metabolites in crossbred dairy cows, and clarify the regulatory mechanism underlying generational differentiation of milk quality traits, so as to provide a theoretical basis for improving milk quality and realizing precision breeding of dairy cows based on hindgut microecology. In this experiment, 10 healthy cows in mid-lactation were selected from each of three consecutive generations (F_1_, F_2_, and F_3_) of Holstein–Norwegian Red crossbred cattle, totaling 30 cows. High-throughput 16S rRNA sequencing combined with UPLC-MS/MS-based untargeted metabolomics was applied to systematically analyze the correlations between the rectal microbial community structure and metabolic profiles and milk quality indicators. The results indicated that milk quality traits exhibited obvious generational differentiation: daily milk yield increased gradually with generation and was significantly higher in F_3_ cows than in F_1_ cows (*p* < 0.05), while milk fat rate and milk protein rate showed an upward-then-downward trend and reached the highest level in the F_2_ generation. The rectal microbial community underwent directional generational succession, with the relative abundance of Firmicutes continuously increased and Actinobacteriota gradually decreased, and the microbial functions were mainly specialized in carbohydrate and amino acid metabolism. Significant generational differences were also observed in rectal metabolic profiles, and the differential metabolites screened between F_1_ and F_3_ generations were primarily enriched in steroid hormone biosynthesis, nucleotide sugar metabolism, bile acid metabolism, and amino acid metabolism pathways. Multi-omics correlation analysis further verified that the core rectal microbial genera could regulate the lactation performance of the mammary gland by affecting the abundance of nutritional metabolic substrates and signaling molecules, with metabolites acting as critical mediators in the regulatory relationship between rectal microbes and milk quality traits. This study systematically characterizes the generational succession characteristics of rectal microbes and metabolites in crossbred dairy cows and identifies potential correlative mediating links of rectal metabolites between microbes and milk-quality traits, providing a novel theoretical reference for exploring hindgut microecology to regulate dairy cow milk quality and assist elite breeding.

## 1. Introduction

Milk and dairy products are rich in high-quality protein, essential fatty acids, and micronutrients. As a core food source for ensuring residents’ balanced dietary nutrition, they occupy a vital position in improving Chinese residents’ nutrition and implementing the Healthy China Strategy [1]. The dairy industry serves as a core pillar for the high-quality development of animal husbandry. The quality of raw milk directly determines the processing added-value and industrial competitiveness of dairy products, and constitutes a key factor restricting the quality improvement and efficiency enhancement of China’s dairy industry [2]. Milk fat percentage, milk protein percentage, lactose percentage, and milk yield are core indicators for evaluating lactation performance and milk quality of dairy cows. Their phenotypes are co-regulated by multiple factors including host genetic background, feeding management, physiological status, and gut microecology [3]. In recent years, with the advancement of multi-omics technologies, the “gut–mammary gland axis” theory has been gradually refined. Gut microbes can remotely regulate mammary gland development and milk component synthesis through pathways such as nutrient digestion, maintenance of metabolic homeostasis, and mediation of immune signals, which has become a research frontier in the field of milk quality regulation [4,5].

Within the gastrointestinal microecosystem of dairy cows, most existing studies have focused on the lactation-regulating functions of rumen microbes, while the roles of hindgut microbes have long been underestimated. As a key functional segment of the hindgut, the rectum conducts secondary fermentation of dietary fiber and polysaccharides incompletely degraded in the rumen, accomplishing crucial physiological processes including short-chain fatty acid synthesis, amino acid remodeling, and bile acid metabolism [6]. Its metabolites can cross the intestinal mucosal barrier into the systemic circulation, target mammary gland tissues, and regulate the synthesis and accumulation of milk fat and milk protein, serving as an important functional carrier of the “gut–mammary gland axis” [7]. Furthermore, gut microbes exhibit considerable host-dependent genetic plasticity and can undergo directional succession along with generational selection, thereby mediating generational differences in lactation phenotypes.

Current domestic and international studies investigating the association between dairy cow microecology and milk quality have largely focused on rumen microbiota analysis within single-generation populations [8,9], highlighting the immediate regulatory effects of microbiota on lactation performance. Nevertheless, the shaping impact of generational turnover on hindgut microbial communities has been generally overlooked. For successive generations of Holstein × Norwegian Red crossbred dairy cows, there is still a lack of systematic multi-omics analysis on the succession patterns of rectal microbiota and metabolic profiles, as well as their intrinsic relationships with generational differentiation in milk quality. Accordingly, this study used crossbred dairy cows from three consecutive generations reared under identical feeding conditions. By integrating 16S rRNA sequencing and untargeted metabolomics, we characterized the generational succession of rectal microbial communities and metabolic profiles, screened core genera and key metabolites governing milk quality, and elucidated the potential mechanism whereby rectal microbes mediate generational differences in milk quality. The results will advance the theory of hindgut microecological regulation of lactation in dairy cows and provide scientific support for precise microecological modulation of milk quality and elite breeding of dairy cattle.

## 2. Materials and Methods

### 2.1. Experimental Animals and Sample Collection

The experiment was carried out at the large-scale dairy farm of Jinhe Dairy Farming Farmers’ Professional Cooperative in Qilihe District, Lanzhou. Experimental animals were Holstein (♀) × Norwegian Red (♂) crossbred offspring with consistent parity (parity 2–3), good health status, and in mid-lactation (120–150 d in milk), which were raised under uniform feeding and management conditions throughout the trial. Ten cows were selected from each of the F_1_, F_2_, and F_3_ generations. During the experiment, all cows were fed total mixed ration (TMR) with identical composition, with ad libitum access to feed and water, and individual daily milk yield was accurately recorded every day. At the end of the trial, fresh rectal digesta were collected under aseptic conditions. Samples were aliquoted into sterile cryotubes, immediately snap-frozen in liquid nitrogen, transported to the laboratory, and stored at −80 °C for microbial DNA extraction and metabolomic analysis. Meanwhile, fresh milk samples were collected from each cow at morning, noon, and evening milking. Equal volumes of subsamples were pooled, and 100 mL composite milk sample was taken, stored at 4 °C, and assayed for milk quality parameters within 24 h.

### 2.2. Milk Quality Measurement

The milk fat percentage, milk protein percentage, and lactose percentage of milk samples were determined using the MILKYWAY-S milk composition analyzer (Zhejiang Luheng Environmental Technology Co., Ltd., Hangzhou, China). Individual daily milk yield was calculated according to daily milking records. All indicators were measured in triplicate, and the average values were used for subsequent statistical analysis.

### 2.3. 16S rRNA Sequencing Analysis of Rectal Microbiota

Total microbial DNA was extracted from rectal digesta using the MN NucleoSpin 96 Soil kit (MACHEREY-NAGEL, Düren, Germany). DNA concentration, purity (A_260_/A_280_), and integrity were examined using a spectrophotometer (NanoDrop 2000; Thermo Fisher Scientific, Wilmington, DE, USA) and 1% agarose gel electrophoresis (instrument details in Supplementary Table S1). The V3-V4 hypervariable region of bacterial 16S rRNA was amplified with primers 338F/806R. Purified PCR products were sequenced on the Illumina MiSeq platform (Illumina, Inc., San Diego, CA, USA). Raw reads were processed for assembly, quality-filtering, and chimera removal (software versions in Supplementary Table S1). Sequences were clustered into OTUs at 97% similarity and taxonomically annotated against the Silva database to obtain phylum- and genus-level community profiles. We calculated alpha-diversity indices (ACE, Chao1, Shannon, Simpson, PD_whole_tree) and generated rarefaction curves. Bray–Curtis-distance-based PCoA combined with PERMANOVA was applied to evaluate inter-group community differences, and PICRUSt2 (v2.1.0-b) was used for KEGG functional prediction (software version in Supplementary Table S1).

### 2.4. UPLC-MS/MS-Based Metabolomic Analysis of Rectal Digesta

Rectal digesta were extracted using methanol-acetonitrile (1:1, *v*/*v*) containing 2-chloro-L-phenylalanine as internal standard, followed by vortexing, ice-bath sonication, −20 °C incubation, centrifugation, vacuum-drying, and reconstitution in acetonitrile-water (1:1, *v*/*v*). QC samples were prepared in parallel to monitor analytical stability. Metabolite profiling was performed on a UHPLC-Q-TOF-MS platform (Agilent Technologies, Inc., Santa Clara, CA, USA) equipped with an HSS T3 column under both positive and negative ESI modes. Progenesis QI v2.3 was used for peak processing and normalization. Metabolites were annotated against METLIN and an in-house database. Differential metabolites were screened using thresholds VIP > 1, FC > 2, *p* < 0.01, followed by KEGG pathway annotation and enrichment analysis (software version in Supplementary Table S1).

### 2.5. Multi-Omics Integration and Data Analysis

Milk-quality indicators were subjected to one-way ANOVA with Duncan’s post hoc test in IBM SPSS Statistics v25; *p* < 0.05 was defined as statistical significance. After dataset quality control, statistical computations and visualization were completed in R software (phyloseq v1.53.0; R-package information in Supplementary Table S1). Procrustes analysis assessed global association between microbial and metabolomic profiles. Spearman correlation was calculated among genus-level differential taxa, differential metabolites, and milk-quality traits. Correlation networks were constructed with cut-offs of |r| > 0.5 and *p* < 0.05.

## 3. Results

### 3.1. Analysis of Differences in Milk Quality Indicators of Dairy Cows Across Different Generations

Analysis of milk quality across three generations of dairy cows revealed that daily milk yield increased significantly with successive generations. The daily milk yield of F3 cows was significantly higher than that of F1 cows (*p* < 0.05). Both milk fat percentage and milk protein percentage showed an initial increase followed by a decrease, reaching the peak in the F2 generation. Values in F2 were significantly higher than those in F1 (*p* < 0.05). Although these indicators slightly declined in F3, they remained higher than in F1. No significant difference was observed in lactose percentage among the three generations (*p* > 0.05) (Figure 1).

### 3.2. Analysis of Rectal Microbial Community Characteristics of Dairy Cows Across Different Generations

#### 3.2.1. Microbial Diversity Analysis

Principal component analysis (PCA) was performed based on the OTU abundance matrix of samples. The results showed that PC1 and PC3 explained 87.45% and 2.23% of the microbial community variation, respectively (Figure 2A). Obvious grouping-aggregation trends were observed among F1, F2, and F3 generations. The Venn diagram showed that there were 1370 shared OTUs across the three groups (Figure 2B). Analysis of inter-group dissimilarity based on Bray–Curtis distance yielded R^2^ = 0.076 and *p* = 0.057 (Figure 2C). The overall inter-group dissimilarity of microbiota was higher than intra-group dissimilarity, which further confirmed a significant differentiation trend in microbial community structure among the three groups. Rarefaction curve results indicated that the curves tended to plateau when the sequencing depth reached 40,000 reads, suggesting that the sequencing depth was sufficient for subsequent analysis (Figure 2D). Alpha-diversity analysis demonstrated that the mean values of ACE and Chao1 indices in the F1 group were higher than those in F2 and F3 groups (Figure 2E,F). No significant difference was detected for the Simpson index among the three groups (Figure 2G). The PD_whole_tree index was significantly higher in the F3 group compared with the F1 group (Figure 2H). The Shannon index presented a trend of F2 > F3 > F1 (Figure 2I).

#### 3.2.2. Species Composition and Screening of Differential Genera

At the phylum level, Firmicutes, Bacteroidota, and Actinobacteriota were the dominant phyla shared by all three groups. The relative abundance of Firmicutes increased significantly across successive generations, while Actinobacteriota exhibited a continuous decrease, indicating obvious differences in microbial community structure among groups. Genus-level analysis revealed that the dominant genus *UCG-005* showed the highest abundance in the F3 group, whereas the abundance of Bifidobacterium gradually decreased with successive generations. Other core genera including *Romboutsia* and *Rikenellaceae_RC9_gut_group* also presented alterations in relative abundance (Figure 3).

#### 3.2.3. KEGG Functional Prediction Analysis

Inter-group differential analysis based on KEGG functional annotation revealed that the relative abundance of core nutrient metabolic pathways, including carbohydrate metabolism and amino acid metabolism, gradually increased along the generational gradient (F1 < F2 < F3). Pairwise comparisons between groups showed that more differentially enriched functional pathways with larger magnitude of differences were observed between F1 versus F2, and F1 versus F3. By contrast, the differences between F2 and F3 were relatively moderate; only a few modules such as carbohydrate metabolism and immune-related pathways exhibited significant divergence (Figure 4).

### 3.3. Analysis of Differences in Rectal Metabolome of Dairy Cows Across Different Generations

#### 3.3.1. Multivariate Statistical Analysis of Metabolic Profiles

PCA revealed obvious separation among samples from the three generations in the score plot, with good intra-group sample clustering. Validation of the OPLS-DA model indicated satisfactory model fitness and predictive power, and no over-fitting was observed. This model was therefore suitable for subsequent screening of differential metabolites (Figure 5).

#### 3.3.2. Screening of Differential Metabolites

Using VIP > 1 and *p* < 0.05 as screening thresholds, the most pronounced metabolic differences were detected between the F1 and F3 groups. A total of 170 significantly differential metabolites were identified, consisting of 56 up-regulated and 114 down-regulated metabolites (Figure 6B). Representative metabolites included PR-toxin, lotaustralin, abscisate, heterodendrin, and 4-hydroxyoct-6-enoylglycine. The metabolic difference between F1 and F2 was moderate, with 42 significantly differential metabolites screened, among which 16 were up-regulated and 26 were down-regulated (Figure 6A). Key metabolites comprised dihydrodeoxy-8-epiaustdiol, GDP-L-fucose, 6-O-galloylglucose, 7-methylguanosine, and PS(16:1(9Z)/0:0). By comparison, the F2 versus F3 comparison exhibited the lowest degree of metabolic variation; only 20 significantly differential metabolites were detected, including 11 up-regulated and 9 down-regulated metabolites (Figure 6C). The differential metabolites included hexamethylene bisacetamide, 7alpha,27-dihydroxycholesterol, (E)-3,7-dimethyl-2,6-octadienyl decanoate, 3alpha,20alpha,21-trihydroxy-5beta-pregnan-11-one, and allylestrenol.

#### 3.3.3. Pathway Enrichment Analysis

KEGG pathway enrichment analysis revealed that differential metabolites between F1 and F2 groups were mainly enriched in pathways including hormone signal transduction, renin secretion, regulation of lipolysis in adipocytes, and multiple nucleotide-sugar biosynthesis. Most of these enriched pathways exhibited an overall down-regulated trend (Figure 7A,D). Comparisons between F1 and F3 yielded the largest number of enriched pathways with the highest enrichment magnitude. The core enriched pathways covered steroid hormone biosynthesis, diverse nucleotide-sugar biosynthesis, β-alanine metabolism, and sulfur relay system. Notably, pathways related to carbohydrate and amino acid metabolism showed particularly prominent down-regulation (Figure 7B,E). In contrast, the metabolic differences between F2 and F3 were primarily concentrated on bile acid metabolism and steroid hormone regulation (Figure 7C,F).

### 3.4. Correlation Analysis Between Rectal Microbiota and Metabolites

Procrustes analysis was performed to assess the coordinated variation between microbial community structure and metabolomic profiles across pairwise group comparisons (Figure 8A–C). A trend of concordant shifts between microbiome and metabolome datasets was observed for all three pairwise comparisons, yet the global associations were not statistically significant.

Further Spearman correlation analysis was performed on differential genera and differential metabolites obtained from pairwise comparisons among the three groups. The results showed that the most pronounced microecological and metabolic discrepancies existed between F1 and F3 groups, involving differential metabolites participating in carbohydrate metabolism, amino acid metabolism, steroid hormone biosynthesis, and secondary bile acid production. By contrast, F1 versus F2 possessed relatively compact core interaction hubs; core fiber-fermenting functional bacteria including Veillonellaceae and Flavonifractor formed strongly positive-correlated modules with key metabolites. For F2 versus F3, only a few Actinobacteriota-derived functional taxa such as Actinomadura and Longisporum exhibited specific positive correlations with phenolic metabolites including 3,4-dihydroxyphenylethylene glycol sulfate (Figure 9).

### 3.5. Correlation Analysis of Rectal Microbiota and Metabolites with Milk Quality

The metabolite-correlation heatmap revealed that a large number of rectal differential metabolites were significantly correlated with lactation and milk-quality indicators. For differential metabolites between F1 and F2 (Figure 10A), most metabolites showed significantly positive correlations with daily milk yield and milk protein percentage. Regarding differential metabolites between F1 and F3 (Figure 10C), the abundances of most metabolites were significantly negatively correlated with daily milk yield. As for differential metabolites between F2 and F3 (Figure 10D), numerous metabolites were significantly positively correlated with daily milk yield, yet significantly negatively correlated with milk protein percentage. Genus-level correlation analysis of rectal microbiota (Figure 10B) demonstrated that Romboutsia was significantly positively correlated with milk fat percentage; Treponema showed a highly significant negative correlation with daily milk yield. No significant correlations were observed between lactose percentage and the majority of rectal genera.

## 4. Discussion

Milk quality variation represents a direct phenotypic manifestation of host genetic improvement, and also acts as the core phenotypic background for gut microecological succession [10]. In the present study, daily milk yield of dairy cows showed a continuous increasing trend with successive generations. The milk yield of F3 cows was significantly higher than that of F1 cows. This is highly consistent with the improvement strategy of “prioritizing yield while giving consideration to quality” during long-term selective breeding of Holstein cows [11]. As a core breeding trait, milk yield achieves continuous genetic progress under directional selection across successive generations [12]. In contrast, milk fat percentage and milk protein percentage exhibited a trend of rising first and then falling, peaking at the F2 generation. Although these two indicators declined slightly in the F3 generation, they were still significantly superior to those of the F1 generation. This is consistent with the metabolic partitioning principle of lactation physiology in high-yield dairy cows [13,14]. Under high-yield conditions, the mammary gland gives priority to lactose synthesis and milk yield output. The synthesis rates of milk fat and milk protein lag relatively behind the increment of milk yield, which finally leads to a slight drop in component percentages.

Accompanied by the changes in milk-quality traits, the rectal microbial communities of dairy cows also exhibited significant characteristics of directional succession. In the present study, Firmicutes, Bacteroidota, and Actinobacteriota were the core dominant phyla in the dairy cow rectum, which was consistent with the reported composition profiles of dairy cow hindgut microbiota [15]. Among them, the relative abundance of Firmicutes continuously increased across successive generations. Core genera including UCG-005, Bifidobacterium, and Romboutsia displayed significant gradient-dependent alterations in abundance. Firmicutes are core functional taxa in the ruminant gut responsible for dietary fiber fermentation, short-chain fatty acid production, and amino acid metabolism [16]. The generational increase in its abundance coincided with the gradual enrichment of carbohydrate metabolism and amino acid metabolism pathways in KEGG functional prediction. This suggests a trend that hindgut microbiota may have enhanced nutrient degradation and utilization capacity under multi-generational selection, which may potentially supply sufficient energy and metabolic precursors for host lactation [17]. Notably, α-diversity analysis showed that the F_1_ generation possessed higher microbial species richness, while the F_3_ generation had markedly elevated phylogenetic diversity. These results indicated that despite a slight decline in microbial richness, community functions were specialized toward nutrient metabolism with broader phylogenetic coverage of functional genes. This represents an adaptive evolutionary outcome of microbiota responding to improved host lactation performance [18]. Previous studies have confirmed that bovine fecal microbiota has moderate heritability and is genetically correlated with lactation performance [3]. Combined with the remarkable microbiota differentiation across the three generations observed in this study, it can be inferred that intergenerational shifts in rectal microbiota are not caused by random drift, but represent a synergistic selective response accompanying the genetic improvement of host lactation traits. Hindgut microbial signatures can serve as potential biomarkers for selecting lactation performance in dairy cows [19].

Structural succession of microbial communities ultimately exerts effects on the host via altered metabolic functions, and the discrepancies in rectal metabolomic profiles are a direct reflection of microecological functional variations [20]. In this study, larger generational intervals gave rise to more pronounced metabolic differences, which was highly consistent with the pattern of microbial succession. Differential metabolites were mainly enriched in three major functional pathways. First, carbohydrate and amino acid metabolic pathways: metabolites such as inositol glycosides and bioactive oligopeptides are not only end-products of microbial fermentation, but also direct precursors for lactose and milk-protein synthesis in the mammary gland. These metabolites may be absorbed across the intestinal mucosa into systemic circulation and transported to the mammary gland, where they are hypothesized to participate in milk component synthesis [21]. Second, bile acid metabolic pathways: bile acid intermediates such as choloyl-CoA act as signaling molecules to activate nuclear receptor pathways including FXR and TGR5, systematically regulating host lipid metabolic homeostasis and thereby indirectly modulating milk-fat synthesis efficiency in the mammary gland [22,23]. Third, steroid-hormone biosynthesis pathways: steroid metabolites can directly regulate the proliferation, differentiation, and lactation activity of mammary epithelial cells through endocrine pathways, acting as vital signal mediators for intergenerational variation in lactation performance [24]. Further comparison revealed that differences between F_1_ and F_2_ were concentrated in nutrient-metabolism substrate pathways, whereas differences between F_2_ and F_3_ shifted toward bile acid and hormone signaling pathways. It is speculated that at the breeding stage with relatively low milk yield, milk-quality improvement mainly relies on hindgut microbiota to raise the supply efficiency of nutritional substrates. When animals enter the high-yield stage, reprogramming of signaling pathways becomes the core driver for milk-component regulation. This pattern provides theoretical references for milk-quality regulation strategies tailored to different breeding stages.

Multi-omics association analysis further validated the regulatory cascade of “rectal microbiota-metabolites-milk quality”. In the present study, functional genera belonging to Firmicutes such as Romboutsia were significantly positively correlated with milk fat percentage. The underlying mechanism may involve the generation of secondary bile acids via 7α-dehydroxylation of primary bile acids by these taxa, which further modulates hepatic and mammary lipid metabolism through the FXR pathway and promotes milk fat synthesis [25]. By contrast, Treponema exhibited an extremely significant negative correlation with daily milk yield. Studies have demonstrated that Treponema is associated with intestinal inflammatory responses; elevated Treponema abundance may trigger low-grade inflammation of the intestinal mucosa, disturb nutrient absorption and metabolic homeostasis, and consequently suppress lactation performance [26,27]. In addition, numerous bioactive oligopeptides and amino acid derivatives were significantly correlated with milk yield and milk protein percentage, directly verifying the substrate-providing role of hindgut amino acid metabolism for mammary protein synthesis [28]. Distinct from the lactation-regulating functions of rumen microbiota, rectal hindgut microbiota is not only involved in secondary digestion of nutrients, but also exerts systemic regulatory effects through signaling molecules including bile acids and hormonal metabolites [29], constituting a non-negligible functional segment within the “gut–mammary axis”. From the hindgut perspective, this study supplements the microecological theory underlying lactation regulation in dairy cows, systematically uncovers the succession pattern of rectal microecology across successive generations, and clarifies the mediating effect of metabolites linking microbiota and milk quality (Figure 11). Nevertheless, several limitations remain in the present study. The interpretation of regulatory relationships in this work relies entirely on correlation analysis, and no functional validation experiments were conducted. Untargeted metabolomics only enables qualitative analysis and relative quantification based on chromatographic peak intensities; absolute quantification using authentic standards was not performed for key differential metabolites in this study, and extrapolation of the findings to other cattle breeds, farming environments, and feeding management systems should be undertaken with caution. Owing to the lack of absolute metabolite concentrations, the practical translational potential of the candidate metabolic biomarkers identified here is limited. Future research should integrate metagenomic sequencing, targeted metabolomics coupled with standard-based absolute quantification, and cell-based functional validation experiments to further elucidate the regulatory mechanisms of core functional bacteria and key metabolites, thereby establishing a more solid theoretical foundation for the development of microecological regulation technologies.

## 5. Conclusions

Significant intergenerational divergence was observed in milk-quality traits of crossbred offspring from Holstein × Norwegian Red dairy cows. Milk yield continuously increased across successive generations; milk fat percentage and milk protein percentage exhibited an initial increase followed by a decline, while lactose percentage remained relatively stable. The rectal microbial communities underwent directional succession over generations. The abundance of Firmicutes as well as the functional capacities of carbohydrate and amino acid metabolism kept increasing, whereas Actinobacteriota abundance gradually decreased. The microbiota was overall specialized toward efficient nutrient utilization, and divergence in community structure was highly coordinated with changes in lactation performance. Meanwhile, rectal metabolomic profiles displayed distinct generational differences. Differential metabolites were mainly enriched in amino acid metabolism, carbohydrate metabolism, steroid hormone biosynthesis, and bile acid metabolism pathways. Alterations in metabolic functions were highly consistent with the succession pattern of microbial communities. In conclusion, core rectal microbes show significant correlations with milk-quality traits, which may be linked to changes in the abundance of nutritional-metabolic substrates and signaling molecules; metabolites exhibit important correlative potential as intermediate factors linking rectal microbial communities and milk-quality traits.

## Figures and Tables

**Figure 1:**
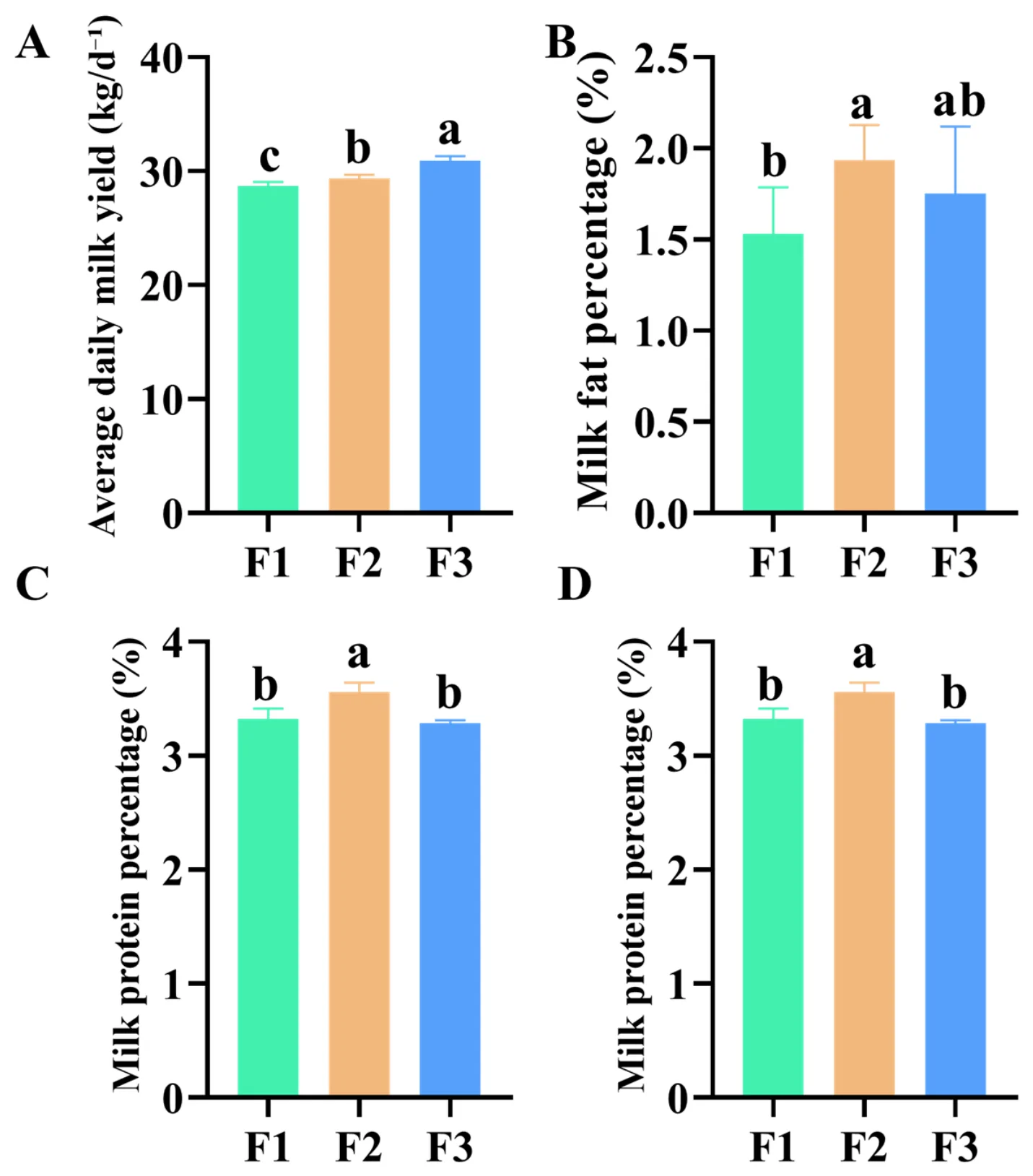
Milk quality indicators of dairy cows across different generations. (**A**): Average daily milk yield; (**B**): milk fat percentage; (**C**): milk protein percentage; (**D**): lactose percentage. Different lowercase letters indicate significant differences among groups (*p* < 0.05). The same notation applies hereinafter.

**Figure 2:**
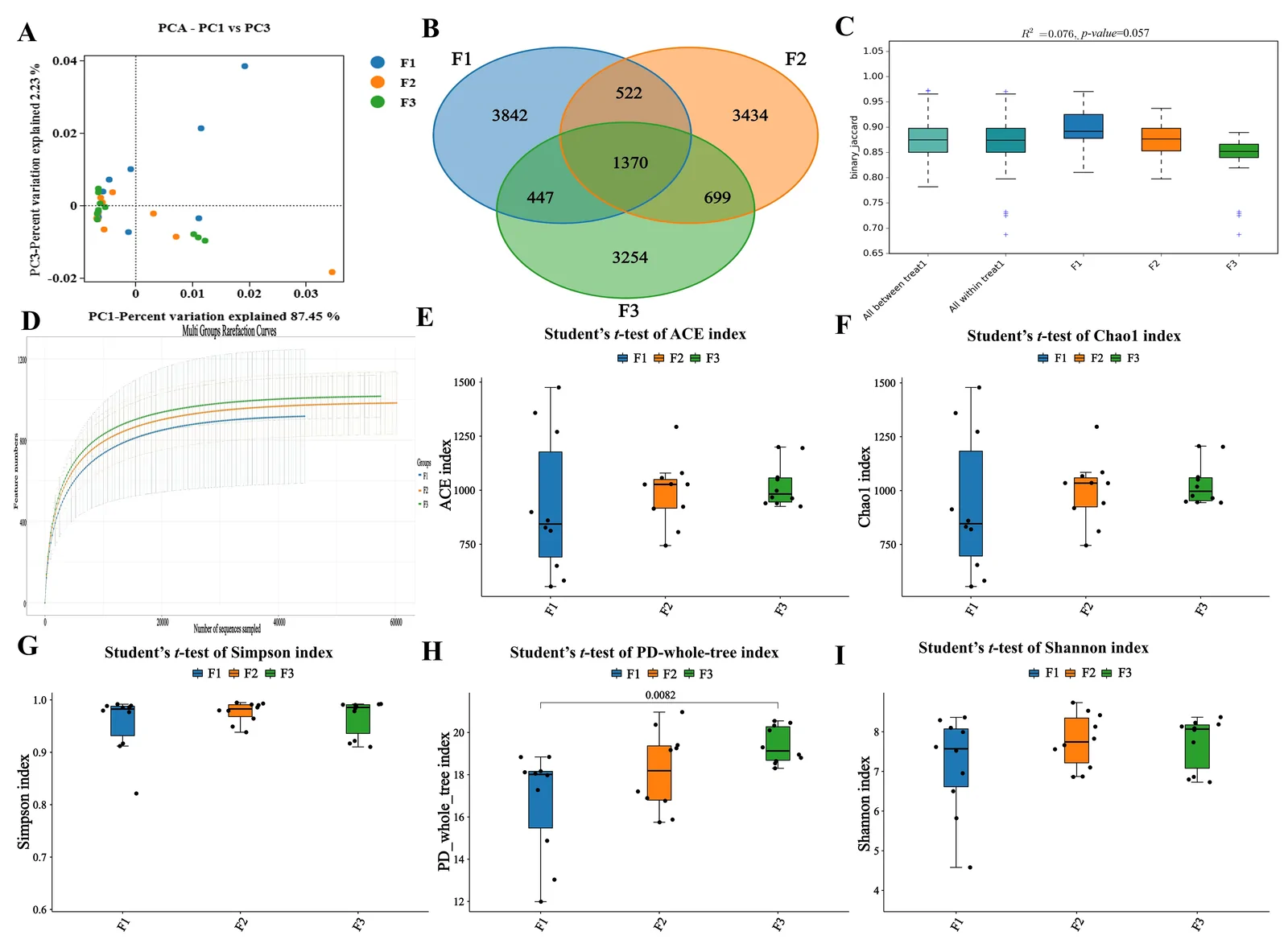
Analysis of rectal microbial community structure and alpha-diversity in dairy cows across different generations. (**A**): Principal component analysis (PCA); (**B**): Venn diagram of OTUs; (**C**): box plot based on Bray–Curtis distance; (**D**): rarefaction curve; (**E**–**I**): inter-group difference tests of alpha-diversity indices.

**Figure 3:**
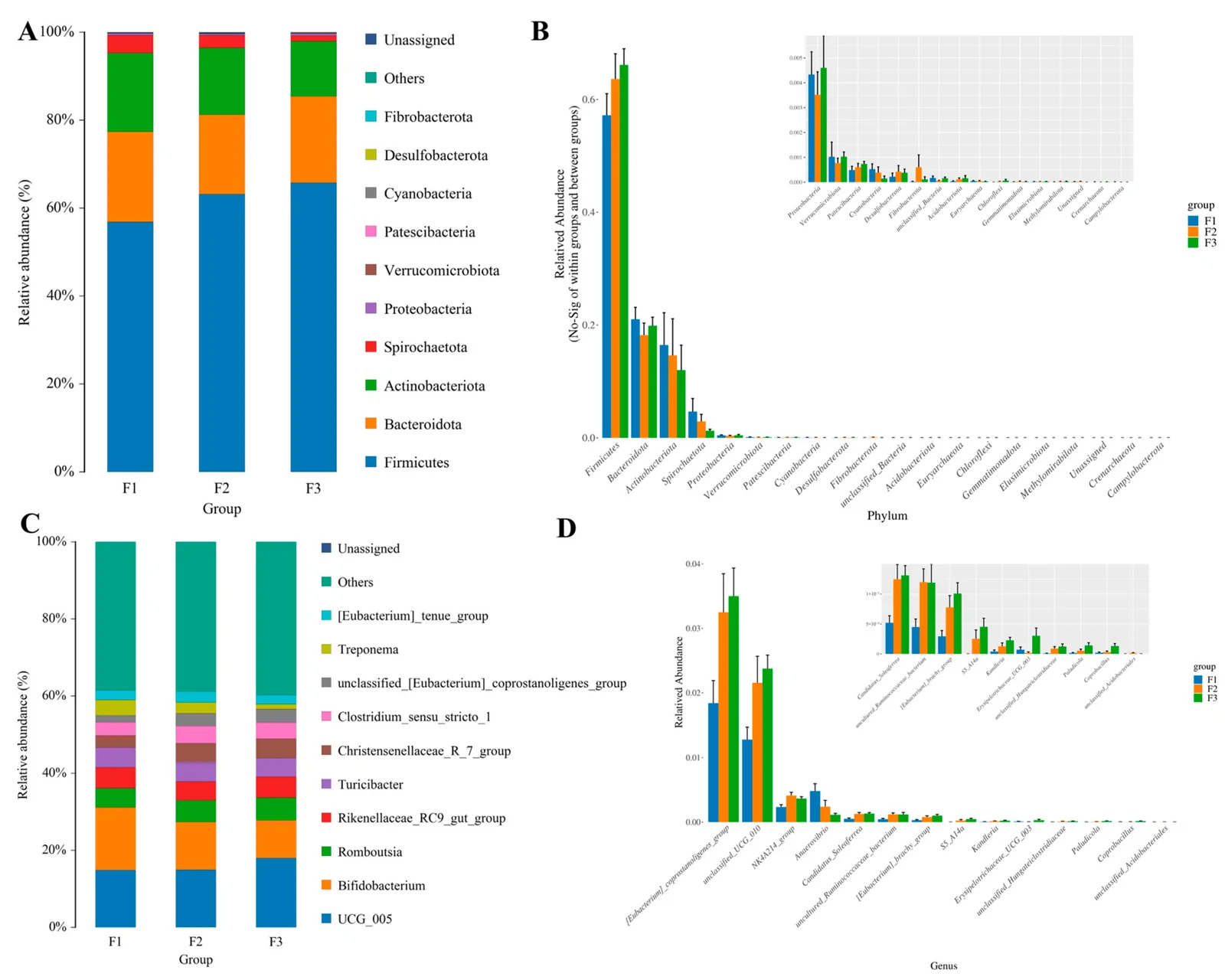
(**A**): Stacked bar chart of taxonomic composition at the phylum level; (**B**): bar chart showing relative abundances of differential taxa at the phylum level; (**C**): stacked bar chart of taxonomic composition at the genus level; (**D**): bar chart showing relative abundances of differential taxa at the genus level.

**Figure 4:**
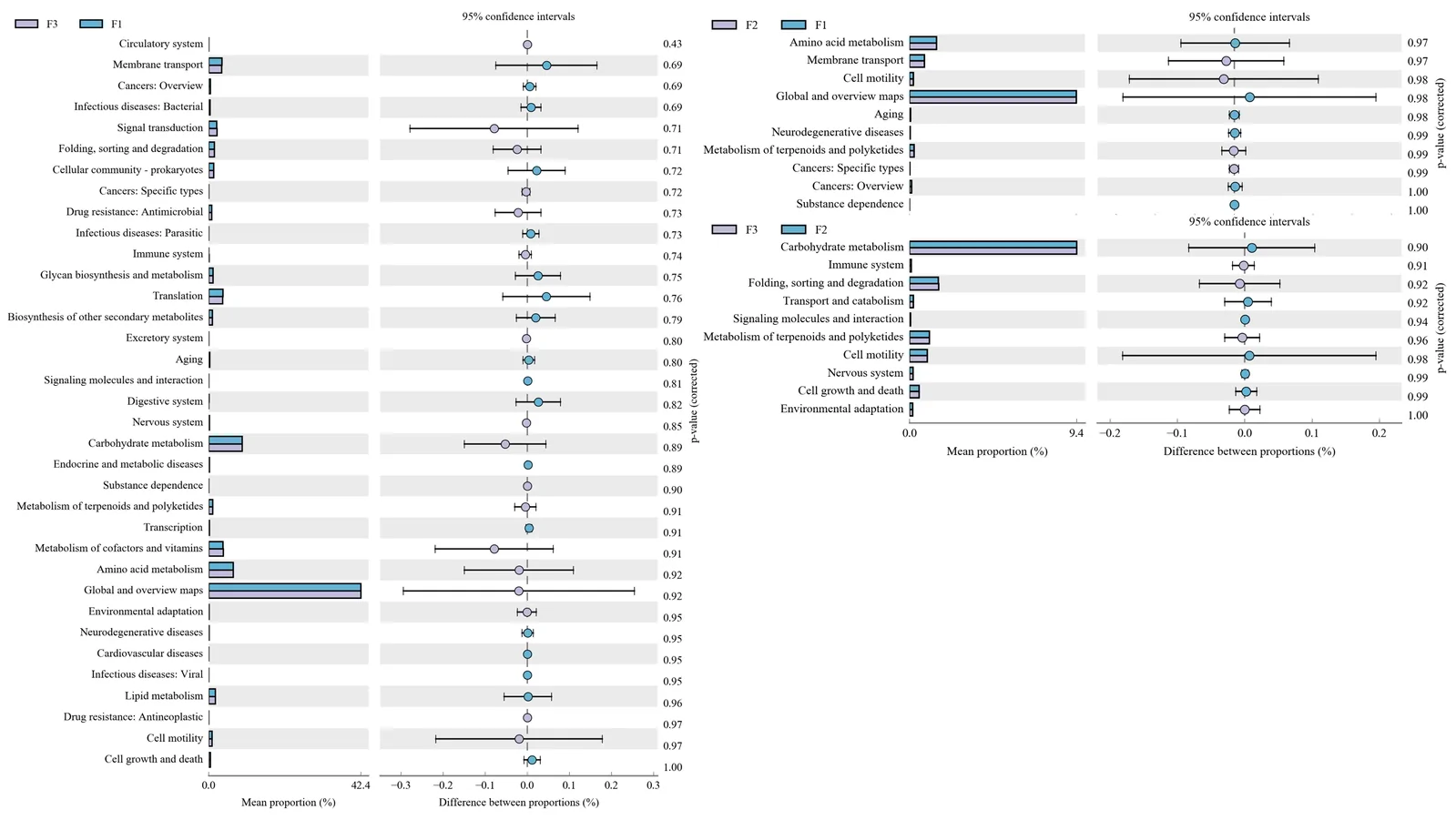
KEGG pathway functional enrichment analysis of rectal microbiota in dairy cows across different generations.

**Figure 5:**
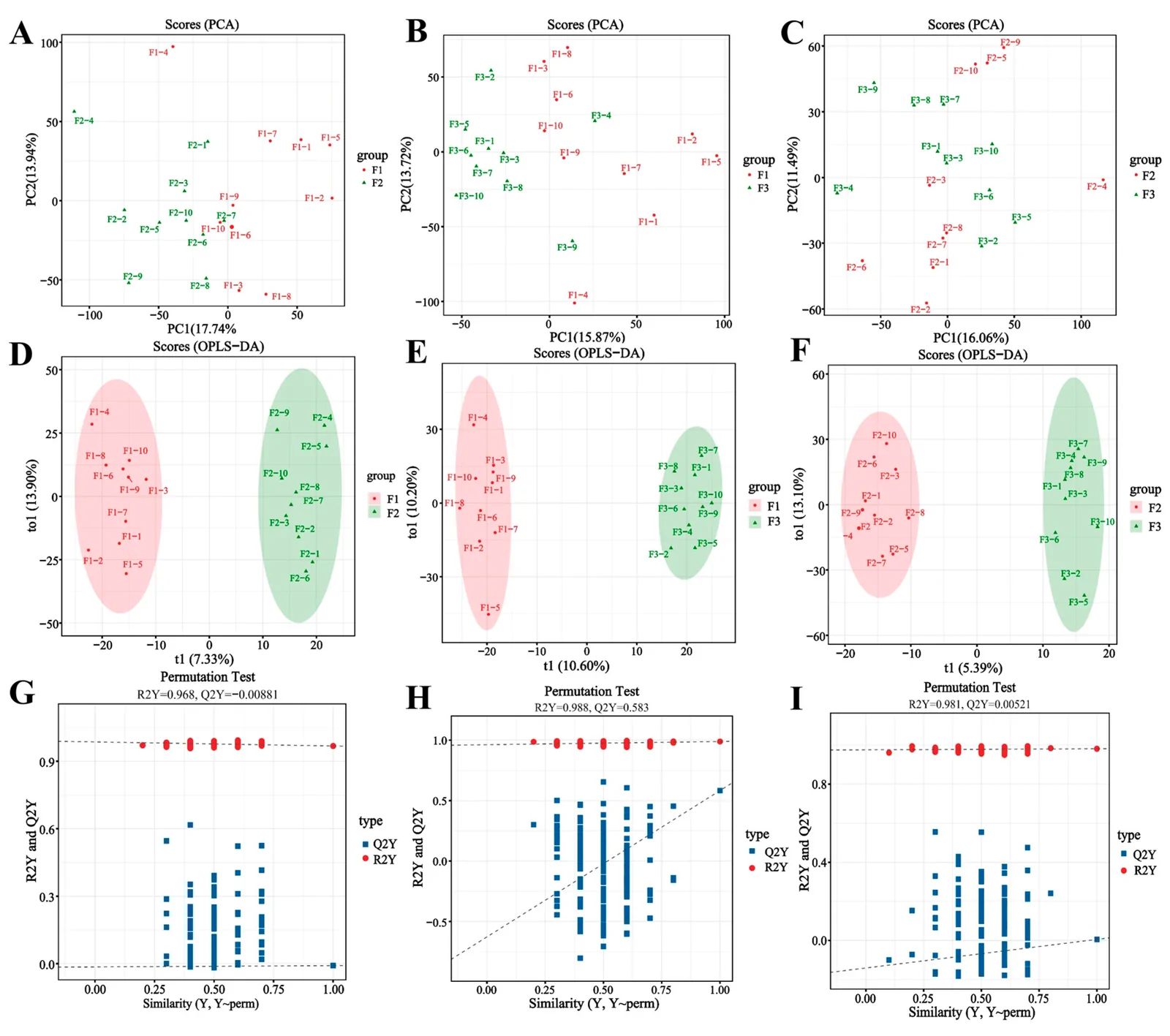
Multivariate statistical analysis results of metabolome among different generations. (**A**–**C**): PCA score plots for pairwise comparisons among three groups; (**D**–**F**): OPLS-DA score plots for pairwise comparisons among three groups; (**G**–**I**): permutation test plots for pairwise comparisons. Red dots represent R^2^Y, and blue dots represent Q^2^Y.

**Figure 6:**
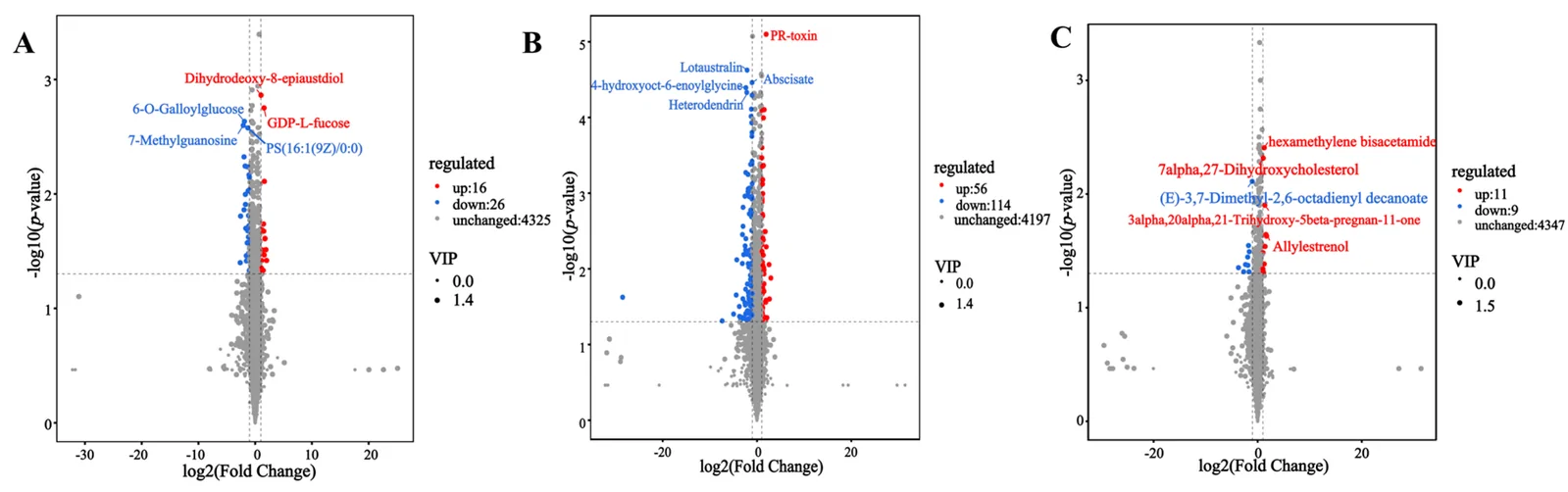
Screening of differential metabolites across different generations. (**A**–**C**): Volcano plots of differential metabolites for pairwise comparisons among three groups. Red dots represent significantly up-regulated metabolites, blue dots represent significantly down-regulated metabolites, and gray dots indicate metabolites with no significant difference.

**Figure 7:**
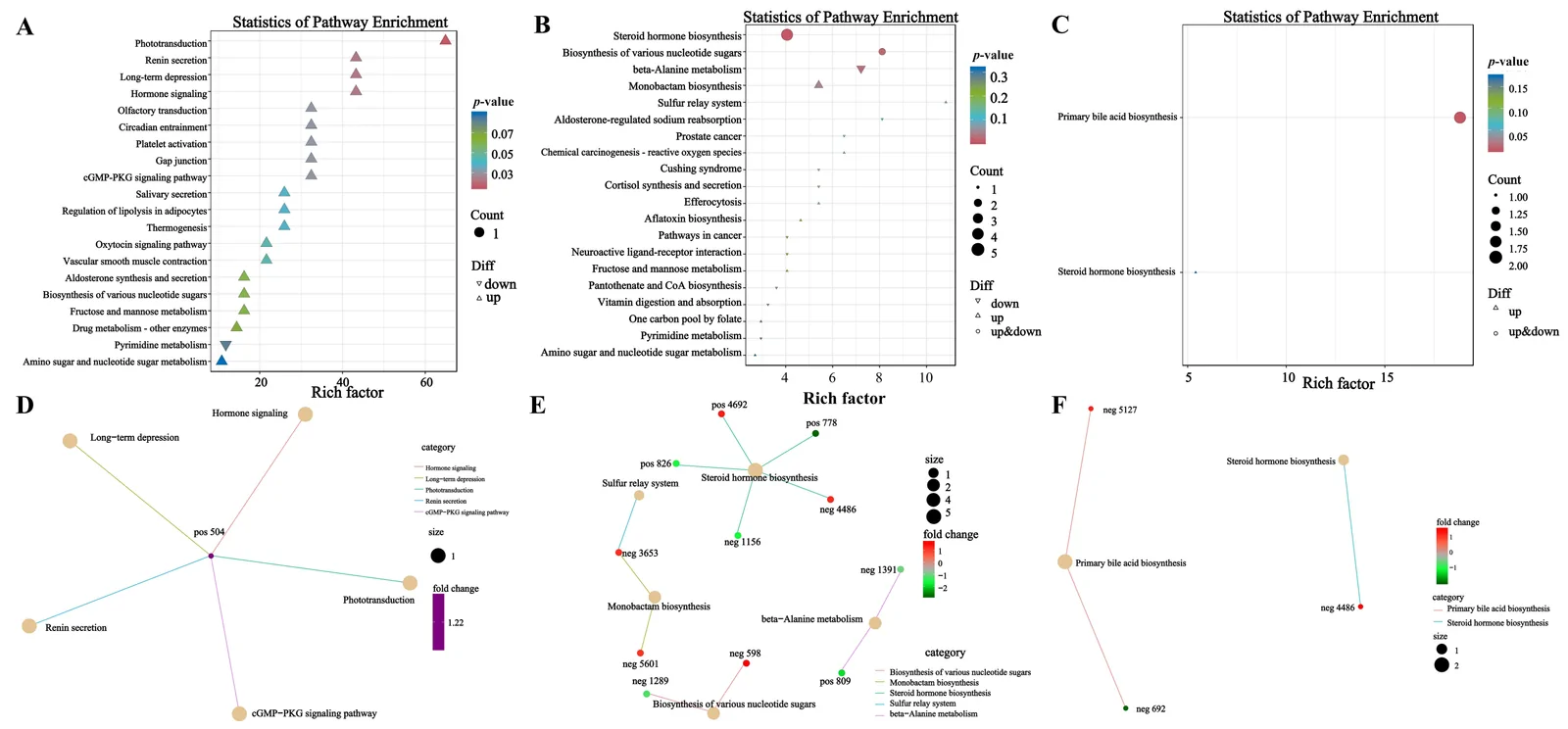
Pathway enrichment analysis of differential metabolites. (**A**–**C**): Bubble plots for pathway enrichment of differential metabolites in pairwise comparisons among three groups. Triangular markers distinguish up-regulated/down-regulated differential metabolites within pathways; (**D**–**F**): correlation network diagrams of key enriched pathways and core differential metabolites for pairwise comparisons among three groups.

**Figure 8:**
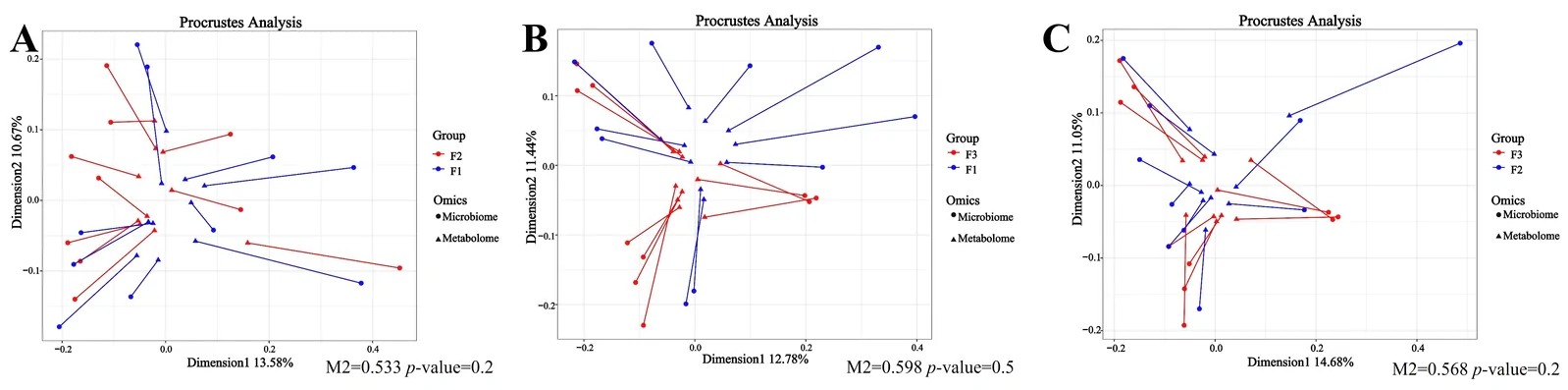
Correlation analysis between microbiota and metabolome. (**A**–**C**): Procrustes congruence analysis plots for pairwise comparisons among three groups.

**Figure 9:**
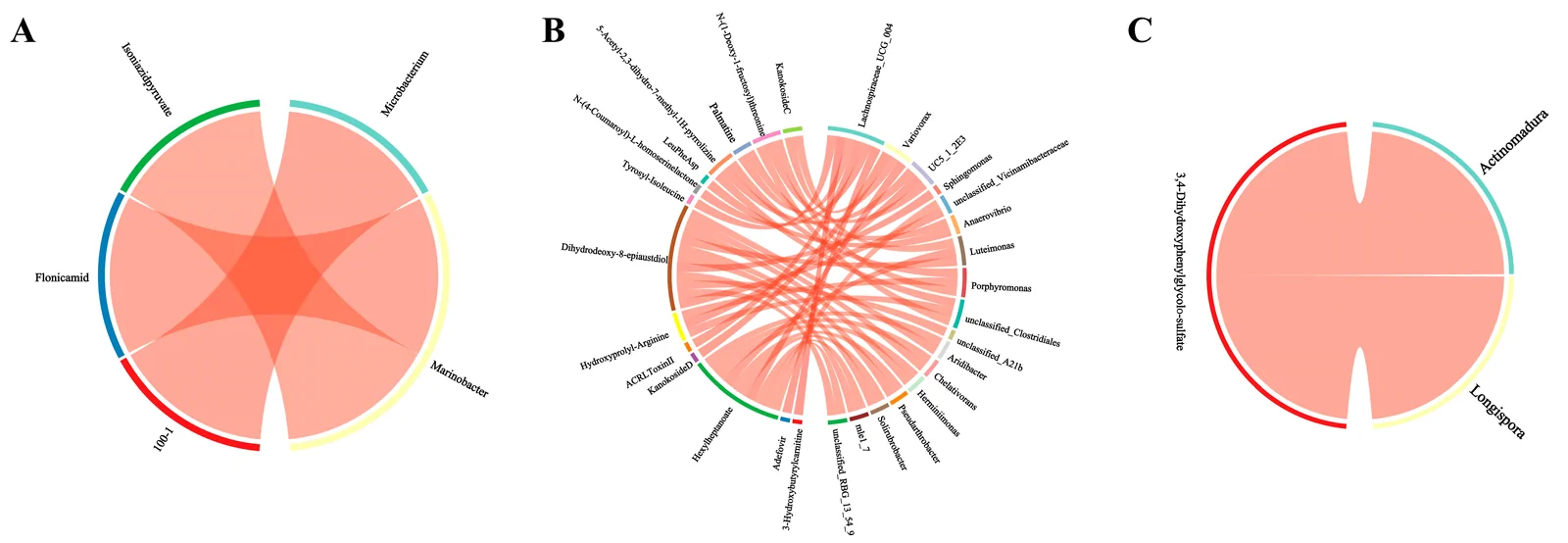
Chord diagrams showing the correlation between microbial taxa and metabolites in three pairwise comparisons. (**A**) F1 vs F2; (**B**) F1 vs F3; (**C**) F2 vs F3. The connecting lines represent significant correlations between microorganisms and metabolites, and the color intensity indicates the correlation strength.

**Figure 10:**
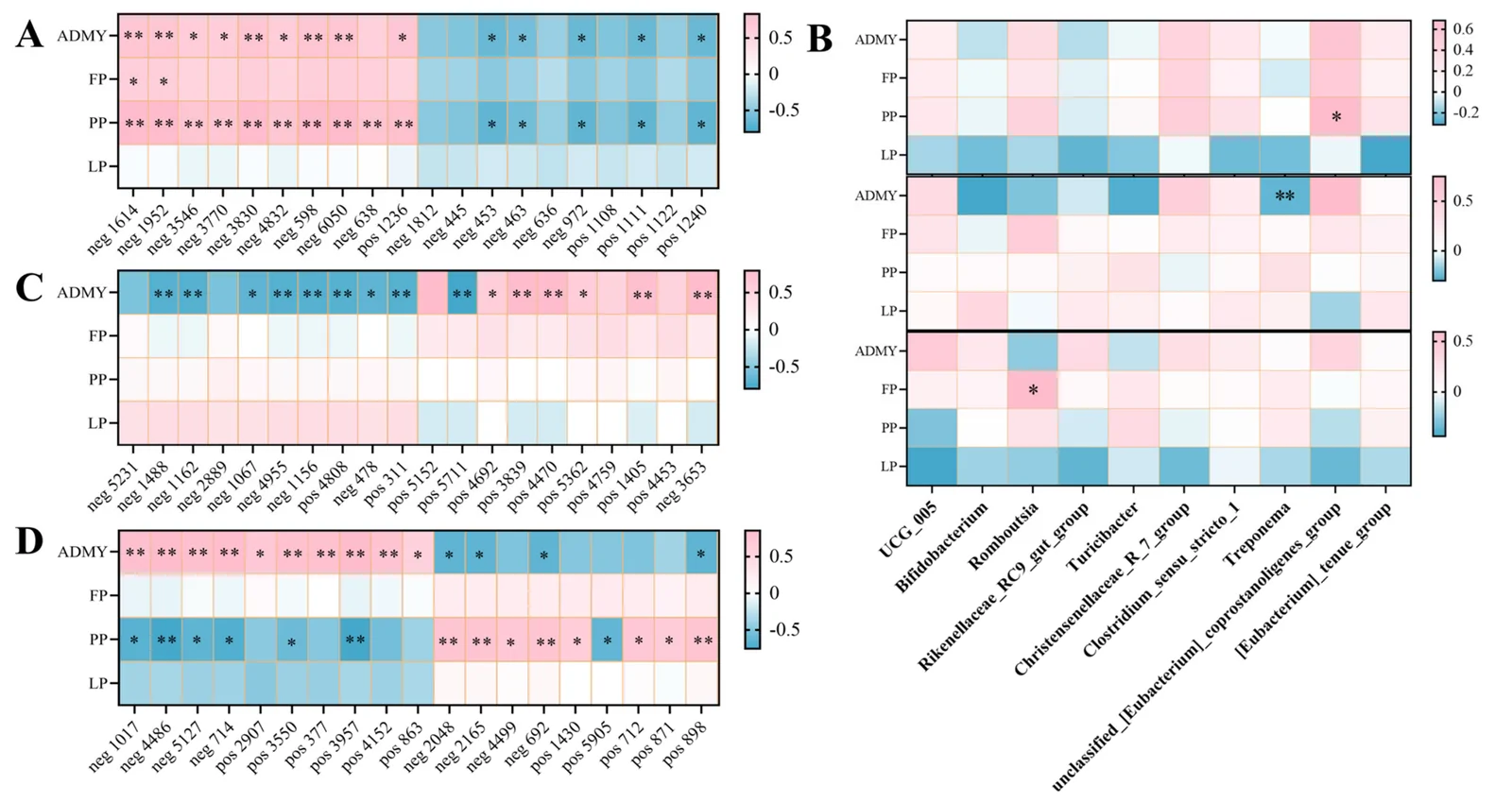
Correlation heatmaps among differential metabolites, core microbial genera, and milk quality traits. (**A**), (**C**), and (**D**): Correlations between differential metabolites and milk quality in F1, F2, and F3 groups, respectively; (**B**): correlations between core differential microbial genera and milk quality traits. Red denotes positive correlations, and blue denotes negative correlations. Asterisks indicate statistical significance: * *p* < 0.05, ** *p* < 0.01.

**Figure 11:**
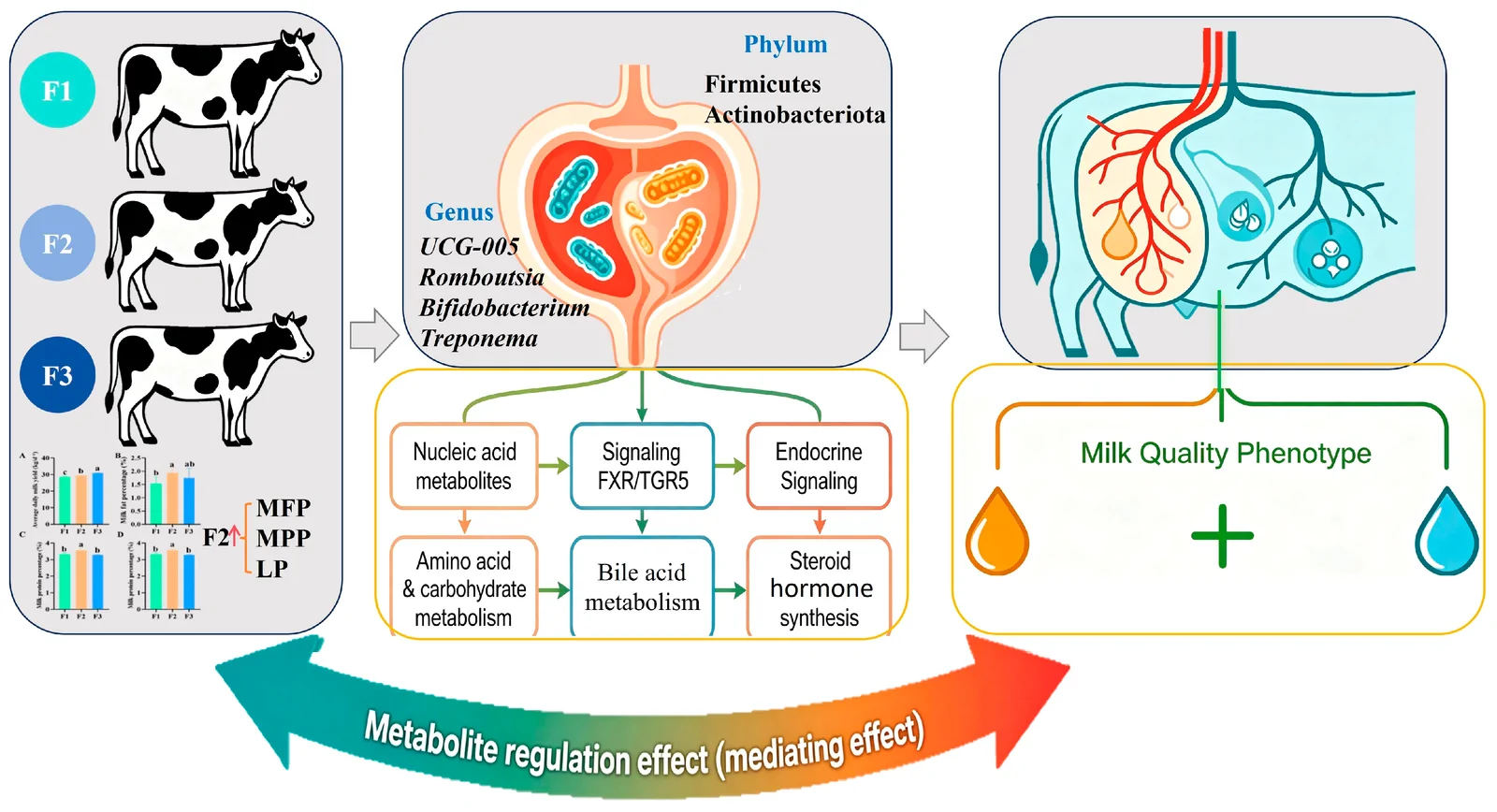
Abstract schematic diagram of the regulatory mechanism underlying generational differences in milk quality mediated by rectal microbiota–metabolite axis in dairy cows.

## Data Availability

The original contributions presented in this study are included in the article/supplementary material. Further inquiries can be directed to the corresponding authors.

## Supplementary Materials

The following supporting information can be downloaded at https://www.mdpi.com/article/10.3390/microorganisms14102169/s1, Table S1: Detailed instrument parameters, software, and version information used in this study.

## Abbreviations

The following abbreviations are used in this manuscript:

OTU	Operational Taxonomic Unit
PCA	Principal Component Analysis
PCoA	Principal Coordinate Analysis
KEGG	Kyoto Encyclopedia of Genes and Genomes
UPLC-MS/MS	Ultra-Performance Liquid Chromatography-Tandem Mass Spectrometry
QC	Quality Control
