# Supplementary material for: Generational Succession of Rectal Microbiome and Metabolome in Holstein–Norwegian Red Crossbred Offspring and Their Regulatory Effects on Milk Quality
Source: Microorganisms. 2026 Sep 25;14(10):2169. doi: 10.3390/microorganisms14102169 (PMC13707599; doi:10.3390/microorganisms14102169)
Supplement: Supplementary file 1 [file microorganisms-14-02169-s001.zip › microorganisms-4580555-supplementary.pdf]

Supplementary Table S1. Detailed instrument parameters, software, and version information used in this study.

Assay	Item	Detail
16S rRNA sequencing	Spectrophotometer	NanoDrop 2000, Thermo Fisher, USA
	Sequence-processing software	FLASH v1.2.7; Trimmomatic v0.33; UCHIME v4.2
	Functional prediction tool	PICRUSt2
Untargeted metabolomics	UHPLC-MS system	Waters Acquity I-Class PLUS UHPLC coupled to Xevo G2-XS QTOF
	Analytical column	Waters Acquity UPLC HSS T3 column (1.8 µm, 2.1 mm × 100 mm)
	Mobile phase	A:0.1% formic acid-water; B:0.1% formic acid-acetonitrile; injection volume 1 µL
	MS acquisition	MSE mode; low collision energy 2 V; high collision energy 10-40 V; scan cycle 0.2 s
	ESI source setting	Capillary voltage: 2500 V(+)/−2000 V(−); cone voltage 30 V; source temp 100 ℃; desolvation gas temp 500 ℃; desolvation gas flow 800 L/h; m/z 50-1200
	Peak-processing software	Progenesis QI
Statistical analysis	Software	R software (version; key packages listed)
